# Clinical Lectures on Scriveners’ Palsy

**Published:** 1865-06

**Authors:** Samuel Solly

**Affiliations:** Senior Surgeon to the Hospital


### CLINICAL LECTURES ON SCRIVENERS' PALSY.

### DELIVERED AT ST. THOMAS' HOSPITAL.

By SAMUEL SOLLY, Esq., F.R.S., Senior Surgeon to the Hospital.

### LECTURE II.

Gentlemen:—Again returning to the subject of scriveners' palsy, I will relate another case, as it illustrates the main characteristics of the disease very forcibly, and also shows that the malady is more easily cured when detected early.

Mr. Q——, aged twenty-five, single. Parents are alive and healthy, and, on his father's side, very long-lived; he has a grandmother aged ninety-two. His habits have always been regular and steady, taking beer with his meals, but not spirits. He smokes very little now, and did not smoke at all when he was first attacked. He has been engaged in mercantile business since he was fourteen years of age, but previously to going into the —— Bank (which he did about five years ago), he was in a large wholesale house. In this house he had less writing, and more active exercise. After he had been in the service of the bank about a year, he complained of an uncomfortable sensation in the upper arm of the right side, but not at first in the hand and fingers. This discomfort was succeeded by violent trembling in the muscles and tendons of the hand and arm, accompanied by a sensation of extreme fatigue in the limb, as if he had been using some violent physical exertion, rendering the arm and hand completely numbed. This numbness was so complete that he could pinch and prick the fingers without feeling anything. At last it became so bad that he could not hold his pen. When these symptoms first commenced they would all retire at night, and, with rest and sleep, he would feel pretty well in the morning; but ere long even this relief was denied him, and he commenced business in the morning with some discomfort. When, at last, the handwriting became so bad that he was ashamed of it, he sought my advice.

I recommended a complete change of air and scene, which, through the great kindness of those who claimed his services, he was enabled to effect. After the lapse of two months away from his writing occupation, during which time he was benefiting by the bracing sea air and bathing at Brighton, he almost entirely recovered. Since that time (beyond an occasional return of the symptoms in a mild degree, after using the pen without cessation for any lengthened period) he has not experienced sufficient inconvenience to lead him to fear a return of the disease. He has not so much writing to do now as formerly, and writes an excellent hand without difficulty.

The next case does not tell much for our medical treatment of such cases, though it shows that with rest they will recover. I have no doubt that the pen he speaks of was of service, but I do not believe that it would have effected a cure if it had been alone employed in the first instance. Coming after the rest, the pen was certainly of use. This patient shall tell his own story in his own words, which are:—

"I entered the service of the —— Bank in November, 1859. About the middle of 1860, I began to experience an involuntary pressure of my thumb upon the pen while writing, which increased until it was so violent as to be very painful, and I was compelled to hold the pen with my fingers, without using the thumb. The thumb then doubled itself into the palm of the hand causing much pain. These sensations were experienced only while writing, or when holding any article between the finger and the top joint of the thumb. At this time I was at

——, in Essex, and sought the advice of a surgeon, who gave me a liniment; but this not proving efficacious, I went to another, who also gave a liniment, and advised me to hold my arm under a stream of cold water, and have it rubbed with the hand in a brisk manner. This was done twice a day for some time. In November, I was removed to the head office, and I cannot remember whether it was before or after this that my thumb so far improved that I could hold the pen with my fingers without the contraction of the thumb upon the palm before complained of.

"On 21st Feb. 1860, I sought your advice in the matter, when you advised me to write as little as possible, and gave me a prescription (this was the third of a grain of strychnine), stating my case to be an attack of the scriveners' palsy, in a slight degree. On the 25th, you increased the dose (to the sixteenth of a grain), and directed me to leave off writing for a fortnight. This medicine caused twitchings of the muscles of the face, and on April 5th, you gave me another prescription (the twelfth of a grain of the bichloride of mercury). On the 16th of April, you advised me to try galvanism, and, by the kindness of Mr. Wood, of Cheapside, I was allowed to go to his shop and have the use of one of his instruments for a fortnight to try its effect. At first it appeared beneficial, but the effect was not permanent.

"During this time I had been writing with my left hand, but I began to feel an unpleasant sensation in my left arm, and you advised me that I must discontinue it.

"On May 9th, you gave me small doses of colchicum, and on the 28th, tincture of iron. The latter, however, I did not use, as I left the bank on the 31st, and went to my home in the country.

"In July, I started on a voyage to the Canary Islands, the journey out and home occupying six weeks. This did not appear to produce any especial good at the time, but I think it had a beneficial effect upon my general health.

"I re-entered the service of the bank in September, taking a post requiring but little writing; and in the same month I attended an orthopædic hospital, and was seen by Mr. —— and Mr. ——. One of these thought the extensors were withered, but the other differed from him. I was put under the care of Mr. ——, and various trials were made of a spring fixed on a glove, intending to prevent the thumb from contracting. But no success attended these efforts, and in Jan., 1862, I ceased my attendance.

"In February, I purchased Perry's 'Orthodactylic' penholder, the peculiarity of which consists in three small plates affixed to the holder in the places where the thumb and fingers should rest. About this time my thumb rapidly improved, and in March I was able, partially, to resume my duties at the books. In April, 1863, I re-entered upon full duty at the desk, since which time I have experienced no inconvenience from the usual labors of the office. (He now writes an admirable hand.)

"A medical gentleman suggested to one of the clerks that the disease might, perhaps, be caused by the contact of the thumb with the steel pens which are used in the office (Mitchell's barrel pens); and since then I have used a quill over the barrel of the pen as a protection, and others of the clerks tell me they have derived benefit by doing the same.

"*Note.*—My habits have always been quiet and regular; no smoker; a teetotaler. In youth I grew fast, and was not of very robust health, though not subject to very severe illness. My father was afflicted with nervousness; mother healthy.

"C. W——, Dec. 14th, 1864."

Very little has been published on the pathology of this disease in the English language. Virchow,* in his handbook of special pathology and therapeutics, has an article upon it; but this, unfortunately, throws no light upon the treatment of this formidable disease. He confirms my opinion that the act of writing is dependent on the co-ordination of several muscles; saying, "Notwithstanding the combinations necessary for this purpose become, by degrees, so habitual that a conscious separation of the single acts is not possible to the mind, it appears as if the rythm of it, as it is learnt with difficulty, yet it in no way approaches the proper automatic movements—such as breathing, &c. Writing requires always a constant attention, which becomes more or less mechanical." This author also refers to an irritability of the upper dorsal region of the spine, sometimes induced by over-practising on the pianoforte, as indicated by pain in that region, and morbid contractions of the fingers. I have seen such cases, and may mention, as a digression from our consideration of its pathology, that I always prescribe nervine tonics, and rest from that kind of action of the muscles—but not entire rest of the limb; active exercise in the open air, with a hoop where it is possible, and calisthenic exercises of all kinds.

This aching in the spine may also be observed in connection with scriveners' palsy; and the fact is important, as I shall explain further on. Very little consideration of the subject of

* Virchow: Handbuch der Speciellen Pathologie und Therapie, vol. iv., p. 143.

scriveners' palsy is needed to convince us that the disease is not a simple paralysis of muscular power. The patient can call all his muscles into action; but he cannot bring them all into such *harmonious* action as to be able to write. What is the reason of this? I must not, even in a clinical lecture, take it for granted that you are all well up in the physiology of the brain and spinal cord, and I am sure I need not ask forgiveness of those who are, if I go a little into the A B C of the matter.

The physiological and pathological explanation of this disease is not given by Virchow. It must be sought in the newest discovered mines of nervous lore. I believe that I was one of the first to insist upon the distinct office of the cineritious and medullary neurine, and I felt that this was the more necessary, inasmuch as Gall and Spurzheim, those grand pioneers in nervine anatomy and physiology, omitted to adopt this view. They merely spoke of the cineritious neurine as the womb or producer of the white, not, as is now universally acknowledged, the real and sole source of power. Much has been discovered lately in regard to the physiological action of this grey neurine—the vesicular portion of the nervous system, and its relation to the tubular, especially as regards the spinal cord. The spinal cord, as I suppose most of you know by this time, is not merely a large nerve or large bundle of nerves—in other words, it is not merely a conducting instrument. It consists of the two kinds of neurine, the tubular or conducting neurine, and the vesicular or dynamic neurine; the white or tubular neurine, the grey or vesicular neurine. I remember when Marshall Hall first enunciated his views and discoveries regarding the especial power of the spinal cord in the production of what he called its excito-motory power, I said, "Well, if you are right, the instrument of that power is the grey matter of the cord." To which he replied, "I know not at all what is the instrument of that power, or in what part of the cord it is developed; I only know of its existence, and that I have proved." Since then the grey matter has been proved to have that function, but it has also been proved to have another distinct from it. It is this power and the machinery of it, so to say, that you must understand, if you do not do so at present, before you can understand what I believe to be the physiology and pathology of scriveners' palsy.

Ever since the days of Hippocrates, it has been known that if the fibres of the brain are torn through by an irruption of blood into their substance, the communication by which the will flows to the muscles is cut off, and the muscles supplied by such fibres are paralyzed. Until a very late period it was supposed that the cerebrum and the tubular fibres of the spinal cord were the only instruments concerned in producing the action of muscles. Latterly, however, the physiologist has felt convinced that there must be some additional instrument concerned in the transaction; some instrument for co-ordinating or combining the action of muscles. The cerebellum has now, by almost universal accord, a portion of this power attributed to it. Schroeder Van der Kolk does not consider that all the credit is due to the cerebellum, but that the vesicles of the cord also have their share in it. I confess that I think so too. The exact proportion in which this power of co-ordinating muscular action is possessed by these centres, I suppose no one will at present pretend to decide. I expect that morbid anatomy, unveiled by the microscope, will clear up a great deal that is yet obscure. Romberg, who gives a short notice of writers' cramp in his work,* mentions a fact which shows that it may take its origin in the cerebellum:—"Paralysis of the upper extremity, dependent on a cerebral or spinal affection, frequently commences with impaired power of conduction in the motor nerves of the fingers, and consequent difficulty of writing. A man was under my care, whose disease lay in the cerebellum, and made its *debut* with an impairment in writing." It will only be by the accumulation of such facts that the mystery will be unveiled.

In the present state of our knowledge of the symptoms which attend scriveners' palsy, I think we are justified in believing that the disease commences in the cord. We find that most of the patients have felt some uneasiness in the lower part of the cervical region, and in the upper part of the dorsal. There is seldom any complaint of discomfort in the head, either in the occipital region (the seat of the cerebellum), or in the frontal. Still it must be allowed that the whole question of its pathology is very much in the dark, though I hope before long a *post mortem* examination may help us out of our dilemma. Without, therefore, now attempting to decide whether the nervous power which co-ordinates the muscles of the writer resides in the cerebellum, or in the spinal cord—though I am inclined to favor the latter hypothesis—we will consider the nervine arrangement by which it is in all probability produced; and though I shall describe it as existing in the spinal cord, still there is no reason to doubt that to a certain extent the same physical arrangement exists in both dynamic centres.

* A Manual of the Nervous Diseases of Man. By M. H. Romberg, M.D. Translated by Dr. Sieveking, for the Sydenham Society, vol. i., p. 321.

Lockhart Clarke and Schroeder Van der Kolk were, I believe, the first to explain clearly the nervous mechanism, if I may so express it, by which a group of muscles are made to act in such unison that complicated actions are produced in perfect harmony. The first-named gentleman originally demonstrated the actual connection of the nerve-tubules with the nerve-vesicles. Schroeder Van der Kolk followed it up and reasoned upon it.

We are so accustomed to see all muscular actions harmonious, that we neglect to consider the means by which this result is obtained. Let us consider the act of volition, and how it is carried out. I will state the matter briefly and dogmatically. Will springs from the action of the hemispherical ganglion, or cortical substance of the hemispheres of the brain. It descends along the white fibres of the hemisphere through the corpus striatum, crura cerebri, pons Varolii, and medulla oblongata, where it crosses from one side to the other, and then descends in the cord to the vesicular neurine in the interior of the medulla spinalis. Now I must have recourse to a diagram on the black board. You will see by this diagram that I represent a nerve-fibre (*a*) coming down from the brain, joining the nucleated vesicles (*b*), those ministers of power in the substance of the cord, the cineritious neurine. I think it right to repeat these terms frequently, in order that they may be household-words, engraved on the tablet of your memory. From these vesicles you see other and more numerous nerve-tubules (*c*) arising; these go to all the numerous flexors and extensors of the hand, which produce the act of writing. If the nerve-tube (*a*) went directly from the cord to the muscles of the hand, those muscles would act, but they would act spasmodically, not harmoniously. The instrument then which produces all this harmony, which co-ordinates the muscular action necessary to writing, whether good or bad, are the nucleated vesicles marked *b*. It is no sound argument against this theory that the act of writing does not come by instinct like the acts of the lower animals, but that the child has to learn to write—in other words, that these vesicles have to learn their co-ordinating duties. Is it not so with the brain itself? The child cannot reason and think accurately as soon as it is born. The ganglionic cells of the hemispherical ganglia have to be educated just as much as any other part of our living mechanism.

The observations of Schroeder Van der Kolk, to which I have referred, are given by him in the work published by the Sydenham Society, and translated by Wm. Davis Moore, Esq.,

M.B.* As I hope you will all take the first opportunity of reading this admirable treatise, I shall not give the author's words at length.

The main point on which you should have a clear idea is this: That the nerve-fibres which you trace with your dissecting knives into a muscle or group of muscles, do not spring directly from the brain, or run without interruption from it. These nerve-fibres spring from "a group of mutually connected ganglionic cells, which cells receive the impressions of our will along the anterior white columns." These impressions or stimulations being thus distributed uniformly over all the cells of a group, produce in all the motor filaments of a nerve arising from this group a uniform and simultaneous action.

The number of these anterior conducting filaments of volition must thus be proportional to the number of the group of cells, and the several combinations of which they are capable. The greater size of the anterior grey cornua in the cervical and lumbar enlargements of the spinal cord are accounted for by this fact.

If we accept this view of Schroeder Van der Kolk of the anatomy and physiology of the spinal cord, confirmed as it is by the earlier and later descriptions of Lockhart Clarke, we are, I think, justified in believing that the writers' palsy must depend upon the disturbance or actual disease of these spinal cells. I must not, however, enter upon this part of the subject in this lecture, but reserve it for my next.

### LECTURE III.

Gentlemen:—Trusting that you have not forgotten what I told you in my last lecture, regarding the anatomy of the spinal cord, its groups of cells and nerves connected therewith, I will now endeavor to prove to you that these cells are frequently diseased, and that upon such disease, in all probability, the scriveners' palsy depends. Scriveners' palsy is not, so far as we know, a fatal disease; patients do not die from it, but they may die with it, and as we have not yet any recorded case of a *post mortem* after scriveners' palsy, I want you to look out for it. Nevertheless, it has been proved that these ganglionic cells are liable to disease—a disease which in its early stages cannot be recognized by the naked eye. The microscope must aid us. Thin sections of a healthy cord show all the beautiful anatomy, which my old pupil, Lockhart Clarke, has so per-

* Professor S. Van der Kolk on the Minute Structure of the Spinal Cord and Medulla, &c., p. 81.

fectly elucidated, that the Royal Society have awarded him the Royal Medal. In health, the microscope shows the vesicles plump and transparent, with a distinct nucleus and nucleolus. In disease, the microscope shows the vesicles no longer transparent, but more or less filled with granular matter, and the nucleus more or less obliterated. In Beale's "Archives," No. XIII, vol. iv., p. 41, you will find from the pen of Mr. Lockhart Clarke, an admirable account of this granular degeneration. Again let me repeat that this description does not apply to a case of scriveners' palsy, and you will, therefore, please to understand distinctly that the account I am going to give you of granular degeneration and granular disintegration of vesicular neurine, as described by Lockhart Clarke, does not apply, at the present time, to scriveners' palsy. If, however, it should hereafter be proved that I am right in my theory, then also will be proved that granular degeneration, in its early stage, may be arrested, and that fresh and healthy cells are reproduced, if these cells are left completely at rest to perfect the reproduction of the cell-structure, and not interfered with during the reproductive action, by attempts on the part of the patient to use the damaged centre of nervous power—in other words, by resting entirely the group of muscles which are called into co-ordinate action by such centres.

The first case in which Mr. Lockhart Clarke made this discovery of granular degeneration of nervous tissue in connection with paralysis, is described in vol. iii. of Beale's "Archives," beginning page 1. The whole paper ought to be, if it has not already been, perused by every practitioner of medicine and surgery. I referred to the facts in my lectures at the College of Surgeons the year before last, and I was surprised to find how many of that learned body of men were unacquainted with the discovery which had been made. It is entitled "An Important Case of Muscular Atrophy, accompanied with Disease of the Spinal Cord." I do not think we shall often see this title again. It implies that the muscular atrophy was the disease, and the condition of the spinal cord the accompaniment; whereas, the disease of the cord was the primary malady, and the muscular atrophy the necessary and invariable consequence of long-continued disease of its co-ordinating centre.

The only grounds upon which pathologists could base the idea of muscular atrophy consequent upon paralysis without disease of the spinal cord has been cut from under their feet by this discovery. Hundreds of such cases have been examined, and reported to be free from disease because the disease was not patent to the naked eye. There is no longer any excuse for such nonsense. No *post mortem* examination of the spinal cord can be considered as conclusive, unless it is examined in thin slices under the microscope.

The important features of the case, and those which appear to me at all to bear upon the pathology of scriveners' palsy are these:—The patient was sixty-five years of age, leading a sedentary life, engaged in literary occupations. "In the end of 1855 he began to complain of neuralgic pains in the balls of the thumbs of both hands, which before long extended to the fore-arms and arms. After some months he was conscious of marked weakness in the hands more especially in the right, soon followed by perceptible diminution of size in the muscles of the thumbs and index fingers, which also became bent inwards and towards the palms, as an habitual posture. All this time, while his general health was good and his body corpulent, he made great complaints of severe neuralgic pains shooting down his arms into the hands and thumbs, and also of weakness of the back.-------Nothing abnormal could be detected in the organs of the chest or abdomen. The kidneys and bowels acted regularly, and apparently naturally; he had no tenderness of the spine, no headache, and his mind, when not turned on his own condition, was clear and rational."

The further history of Dr. P.'s case, up to the time of his death, in January, 1861, (the disease lasted, therefore, a little over five years,) "which was preceded for a few days, and possibly caused, by an attack of acute laryngitis, is nothing more than a detail of the gradual increase in the intensity of the symptoms already noted, and the extension of the pains to the lower limbs and feet, where the sensation of intense heat and burning was constantly complained of, aggravated by the act of walking."

I shall not attempt to give you the detailed account by Mr. Lockhart Clarke of the *post mortem* appearances, but such a very brief abstract as will suffice for my present purpose. There was no disease of the pons Varolii or cerebellum. In a transverse section of the spinal cord, at the origin of the fourth pair of cervical nerves, three lesional spaces might be seen; and this diagram will explain their position. You will recognize the shape of the spinal cord, as shown in a transverse section, and the shape of the grey or vesicular neurine in its centre, like two irregular-shaped letters C placed back to back. The three white spots you see in the grey mass on the left hand are lesional spots, or spots of granular degeneration.

The next case in which Mr. Lockhart Clarke gives us the result of his inquiries, was clearly not a case of scriveners' palsy, but extremely interesting in every point of view. The case is most ably related by Dr. Thudichum, in the fourth volume of Beale's "Archives," page 26, and the brief abstract which I now mean to give you must not prevent your reading the whole account with care and attention.

The patient was forty-five years of age. The cause of disease was concussion of the spine, produced simply by a jump, the patient coming down heavily on his heels. He was stunned at the time, confined to bed for a few days, and then appeared to get quite well. "It was, however, soon perceived that a great change had taken place in his habits. Having been extremely fond of manly sports and exercises—rowing, cricketing, riding on horseback, dancing, and the like—he discontinued to take part in any of them, although he continued to go every autumn to the Scotch moors, for the purpose of shooting grouse." At the age of fifty—*i. e.*, five years after the accident—"while engaged in this latter sport, he perceived that his right leg had lost a part of its usual strength; that although he could perform with it every motion, yet he could not perform it in the manner necessary for the purpose; that he could not put the foot in the exact place where he wanted to put it. In other words, that he could will the action of the muscles of the leg, and that they would obey the will, but, the co-ordinating power being lost, he could not combine their action so as to walk.

Mr. Clarke published in the *British and Foreign Medico-Chirurgical Review*, vol. xxx., p. 219, another interesting case of paralysis, with muscular atrophy and disease of the nervous centres, which Dr. Radcliffe brought to his notice. In this case, he says, "the ordinary and inefficient method of examining the nervous centres must have failed to detect minute structural changes upon which the wasting palsy was actually dependent, and would, perhaps, have resulted in recording the case as one of simple muscular atrophy. In this case, the condition of granular degeneration had gone on to vesicular atrophy. The cells had shrunk; none of them were larger than the *nuclei* of the surrounding healthy cells; the majority were much smaller, without any traces of nuclei or distinct granular contents. These shrunken cells consisted of irregularly-stellate and apparently membranous bodies, which, in some instances, seemed shrivelled sheaths of healthy cells, or like radiating portions of the connective tissue." The accompanying diagram, taken from this paper, shows the relative proportion of the healthy, plump, vigorous cells, and the miserable shrunken sheaths to which by disease they have been reduced.

Amongst other proofs that might be adduced in favor of my view, that the seat of the disease is central in the spinal cord, is the fact that the use of the left hand in writing instead of the right, will induce uncomfortable feeling in the latter. The explanation of which I suppose to be that in writing or drawing the action of the muscles of the left hand, the will having travelled by the conducting fibres of the spinal cord from the brain to the group of cells in the left half of the cord, these cells, on being called into action, excite their neighboring cells, through the medium of the transverse commissure, in the right side of the cord—*i. e.*, those which co-ordinate the muscles in the right, such excitement being manifested by the uncomfortable feeling described. One of my patients, when I spoke to him about the use of the left hand, said he would continue to use it if I wished, but he thought it would be better if he did not use either. He said, "If I write with my left hand, I feel an indescribable tingling feeling in the damaged hand, which does not subside on leaving off writing;" for instance, he continued to feel it whilst talking to me, having been writing half an hour previously. Occasionally he has the same kind of sensation in the right leg. He also says that he has had less of this discomfort since he has held the paper, on which he has to write, by strong clips instead of holding it steady by the right hand, and then, instead of keeping the right hand before him, putting it out of sight behind the body.

Regarding treatment, Virchow says the therapeutical indications to be followed in the treatment of these palsies are very doubtful. "In writers' cramp the application of all means of healing has proved fruitless. Neither narcotics, or stimulants, nor tonics, nor nervines brought any advantage; just as little did counter-irritants, embrocations, electricity, or baths of any sort. Where rheumatism was the cause, Russian vapor-baths, cold douche, electricity, and embrocations with oil of turpentine, were able to remove the complaint. The cutting through of the long muscle of the thumb was tried, with some result, by Stromeyer, in a case in which writers' cramp was conditional on this muscle. Dieffenbach performed the cutting through of the muscle several times, without being able to arrive at anything by it; Langenbeck, with a passing improvement. Nothing therefore remains but the application of different mechani cal appliances which make writing possible. Many such are known, designed by Froschel, Cazenave, and others. One patient helped himself by means of placing the pen in a piece of wood bored through with holes, which he grasped with the whole hand, and so could regulate in writing.

As regards treatment, Romberg (*loc. cit.*, p. 323) says:—"The treatment hitherto pursued, both local and general, has been invariably ineffectual, so that the patients generally ceased from all attempts at cure, and remained satisfied with mechanical contrivances, the object of which was more or less to cause a pressure upon the skin and the subjacent muscles. Stromeyer also applied the principle of division of the muscles to the cure of the writers' cramp, and in one case, a brilliant result justified the anti-spasmodic reputation of tenotomy. As early as the fourteenth day after the subcutaneous division of the tendon of the flexor longus pollicis, the patient was able to resume his pianoforte-playing, and to write. On the other hand, it is to be observed that the operation was perfectly ineffectual in several patients upon whom Dieffenbach operated."

The following passage regarding treatment, in the relation by Drs. Adamson and Bell, of the case of Dr. P——, already referred to, is, I think, instructive:—

"As regards treatment, it may be mentioned that he made a long-continued trial of the following remedies, viz.:—iodide of potassium with sarsaparilla, strychnine, quinine, valerianate of zinc, phosphate of zinc, arsenic, tinctures of lobelia and lupulus, of cantharides, ergot of rye, actea racemosa, with various mineral acids. Throughout the progress of the disease, he employed occasional, and for the last two years regular, preparations of scammony and calomel. He had sedatives in every variety of form, externally and internally, and for the last three years took Battley's sedative solution every night, consuming about thirty ounces each year, besides trying chlorodyne, chloric ether, conium, henbane, lupulin, &c., &c. He tried galvanism and regular friction or shampooing of the limbs, wet bandages, &c. Subcutaneous injection of morphia was repeatedly tried, when pain was concentrated in any individual spot.

"Amongst other systems of treatment, he made a lengthened trial of the waters at Aix-la-Chapelle, and visited also several other German baths, including a water-cure establishment. He also placed himself for a time under a quack doctor, whose grand specific was systematic friction and kneading of the spine. These plans of treatment he discontinued only on being thoroughly convinced that they did him no good.

"Counter-irritation along the spine was also had recourse to, but each and all without the experience of any, even temporary, relief. The diet was always generous, and with a liberal allowance of stimulants."*

No mention is made of complete and entire rest of the palsied hand when the disease first made its appearance. Believing, as I do, that this is the right course to pursue in the treatment of such cases, the fact that the employment of rest was not one of the remedial agents which so signally failed, should encourage us to employ it in any similar case.

My own experience then, in the treatment of these cases is very decided:—rest—entire rest from the occupation that has produced the disease. If the disease is recognized early, then two months' entire rest will arrest its progress; at the end of that time strychnine is of service, but it is injurious when given in the early stage of the disease and before rest. During the time of rest the health should be sustained and improved in every possible way. Fresh bracing air—mountain air if attainable—is of more service than all the tonics in the world. As regards tonics, they must be varied to suit the peculiarities of each constitution. Zinc, iron, and quinine are the tonics on which I rely most.

The following is one of the most striking cases of paralysis I know of, induced suddenly by little more than an hour's continued strain upon a nervous centre, confirming all that I have said regarding the controlling and co-ordinating power of the spinal cord:—A gentleman got into the five o'clock express train for Brighton. He was no sooner seated than he felt a strong desire to have the bowels relieved; but there was no help for it—the train started, nor did it stop until it reached its destination, at a quarter past six o'clock. All this time the desire to go to stool increased more and more, but he restrained it successfully by a violent effort. The moment the train stopped he sprang out of the carriage, when the sphincter ani relaxed, and the contents of the bowels were poured out into his clothes. The curious part of the case is, that he became paraplegic. The spinal cord had been over-exerted, exhausted, and paralyzed by the long-continued effort. In this case the brain, from which I suppose all must admit that the will emanated by which he desired to control his sphincter, did not give way. His intellect was not impaired, and the whole muscular system was not paralyzed, only that portion which is supplied by the lower portion of the cord. I regard this as identical in its pathology to scriveners' palsy, only that the destruction of the nervous centre was sud-

* Beale's Archives, vol. iii., p. 4.

den, not gradual. I do not know what treatment was adopted in this case, but, reasoning from analogy, I should have advised complete rest; that some arrangement should have been made to prevent his ever having to control the action of the bowels if he had retained in any degree that power.

Having told you all I know, or fancy I know, concerning the palsy, and all the speculations I have made regarding the pathological changes produced by, or productive of, the disease, I will ask you to carry on the subject. If you meet with any cases amongst the poor classes of our working-men, send them here, and I will give them a bed, and do the best I can to cure them. If you hear of any such cases—and mind you inquire for them when you go into the country for your Christmas holidays—get your medical friends to watch them; and if they can obtain a *post mortem*, ask them to send me the cervical enlargement of the cord (if they cannot obtain the whole), and we will make sections after the plan recommended by Lockhart Clarke, and test the validity of my views respecting its morbid anatomy. —*London Lancet.*

